# PB.25: Relationship between volumetric breast density, age and hormonal factors

**DOI:** 10.1186/bcr3525

**Published:** 2013-11-08

**Authors:** PF Johnson, JC Sergeant, M Bydder, A Maxwell, S Whiteside, P Stavrinos, M Wilson, DG Evans, A Howell, SM Astley

**Affiliations:** 1 Uaniversity of Manchester Medical School, Manchester, UK; 2 Centre for Imaging Sciences, University of Manchester, UK; 3 Nightingale Centre and Genesis Prevention Centre, University Hospital of South Manchester, UK; 4 Department of Medical Statistics, University Hospital of South Manchester, UK; 5 Department of Genetic Medicine, St Mary's Hospital, Manchester, UK; 6 Institute of Cancer Sciences, University of Manchester, UK

## Introduction

Percentage breast density estimated visually or assessed by computer-assisted area-based measures declines with age, menopausal status and parity and increases with current HRT use. Automated volumetric density measurement methods including Quantra™ and Volpara™ remove subjectivity; it is important to determine how these methods relate to age and endocrine changes and here we describe these associations.

## Methods

Women undergoing routine screening in the NHSBSP who agreed to enter the Predicting Risk Of Cancer At Screening (PROCAS) study completed questionnaires concerning personal information. Percentage volumetric breast density was measured using Quantra™ (21,370 women) and Volpara™ (11,023 women), and WAS related to age and hormonal factors.

## Results

Density significantly declined with age (*P *< 0.001) by both methods. In women aged <50 the mean density by Quantra™ was 19.23 for premenopausal and 16.29 for postmenopausal women (*P *< 0.05), and in women aged 51 to 55 years these figures were 18.06 and 15.72 (*P *< 0.05). For density measured by Volpara™, the corresponding figures are 7.74 and 6.64 (*P *< 0.05) for women aged <50, and 7.54 and 6.00 (*P *< 0.05) for women aged 51 to 55. Current HRT use was associated with significantly higher density (*P *< 0.05) for those aged over 50 (Volpara™) and from 51 to 70 (Quantra™). Nulliparity was associated with higher breast density at all ages (*P *< 0.05 for Quantra™ above the age of 50 and for Volpara™ from age 51 to 65).

## Conclusion

These data indicate that percentage density by both volumetric methods is related to age and endocrine factors in the same directions as area-based methods. It is now important to determine their relationship to risk of developing cancer.

